# A 3-miRNA Signature Enables Risk Stratification in Glioblastoma Multiforme Patients with Different Clinical Outcomes

**DOI:** 10.3390/curroncol29060345

**Published:** 2022-06-16

**Authors:** Vivi Bafiti, Sotiris Ouzounis, Constantina Chalikiopoulou, Eftychia Grigorakou, Ioanna Maria Grypari, Gregory Gregoriou, Andreas Theofanopoulos, Vasilios Panagiotopoulos, Evangelia Prodromidi, Dionisis Cavouras, Vasiliki Zolota, Dimitrios Kardamakis, Theodora Katsila

**Affiliations:** 1 Institute of Chemical Biology, National Hellenic Research Foundation, 11635 Athens, Greece; pmpafiti@eie.gr (V.B.); souzounis@eie.gr (S.O.); cchalikio@eie.gr (C.C.); gregorioug21@stu.acs.gr (G.G.); 2 Biomedical Engineering Department, University of West Attica, 11243 Athens, Greece; eftgrig189@gmail.com (E.G.); cavouras@uniwa.gr (D.C.); 3 Department of Pathology, School of Medicine, University of Patras, 26504 Patras, Greece; iomagry@yahoo.gr (I.M.G.); zol@med.upatras.gr (V.Z.); 4 American Community Schools (ACS), 15234 Athens, Greece; prodromidie@acs.gr; 5 Department of Neurosurgery, University Hospital of Patras, 26504 Patras, Greece; andreastheofano@gmail.com (A.T.); panagiotopoulos2000@yahoo.com (V.P.); 6 Department of Radiation Oncology, University of Patras Medical School, 26504 Patras, Greece; kardim@upatras.gr

**Keywords:** glioblastoma multiforme, 3-microRNA signature, risk stratification, machine learning, image classification, pattern recognition

## Abstract

Malignant gliomas constitute a complex disease phenotype that demands optimum decision-making as they are highly heterogeneous. Such inter-individual variability also renders optimum patient stratification extremely difficult. microRNA (hsa-miR-20a, hsa-miR-21, hsa-miR-21) expression levels were determined by RT-qPCR, upon FFPE tissue sample collection of glioblastoma multiforme patients (*n* = 37). In silico validation was then performed through discriminant analysis. Immunohistochemistry images from biopsy material were utilized by a hybrid deep learning system to further cross validate the distinctive capability of patient risk groups. Our standard-of-care treated patient cohort demonstrates no age- or sex- dependence. The expression values of the 3-miRNA signature between the low- (OS > 12 months) and high-risk (OS < 12 months) groups yield a p-value of <0.0001, enabling risk stratification. Risk stratification is validated by a. our random forest model that efficiently classifies (AUC = 97%) patients into two risk groups (low- vs. high-risk) by learning their 3-miRNA expression values, and b. our deep learning scheme, which recognizes those patterns that differentiate the images in question. Molecular-clinical correlations were drawn to classify low- (OS > 12 months) vs. high-risk (OS < 12 months) glioblastoma multiforme patients. Our 3-microRNA signature (hsa-miR-20a, hsa-miR-21, hsa-miR-10a) may further empower glioblastoma multiforme prognostic evaluation in clinical practice and enrich drug repurposing pipelines.

## 1. Introduction

The ever-increasing development of novel diagnostic tools and targeted approaches is of fundamental importance in the field of oncology. However, glioblastoma multiforme (GBM), the most aggressive type of primary brain tumor, is still associated with poor prognosis and patients’ median overall survival remains limited to 12–15 months [1,2]. According to the European Society for Medical Oncology (ESMO) guidelines for diagnosis, tissue histopathological evaluation is required. The current standard of care is the maximal surgical resection of the tumor followed by radiotherapy with concomitant and adjuvant temozolomide (TMZ), the latter being an alkylating agent [3,4].

As we navigate the big data era, we aim to translate information into knowledge with a primary focus on inter-individual variability. Such a strategy is of paramount importance when brain and central nervous system cancers are considered and this no doubt includes GBM. This approach aims at more precise and powerful diagnostic, prognostic and therapeutic strategies, tailored for each group of cancer patients [5]. GBM is characterized by inter-tumor and intra-tumor heterogeneity, thus the development and validation of potential biomarkers for optimal clinical decision-making becomes extremely demanding. Specifically, the identification of prognostically distinct subgroups of patients, based on common biological backgrounds, may lead to the best clinical outcome through a valuable risk assessment and a well-oriented therapeutic plan [6].

MicroRNAs (miRNAs) are key players in GBM tumor initiation, progression, therapy response and recurrence. The multi-gene targeting ability of a miRNA indicates its function either as oncogenic or tumor suppressor. In this regard, a single miRNA may involve in various cellular processes and pathways, consequently in tumor pathophysiology [7]. Several studies have focused on multiple miRNAs that exhibit distinct expression profiles in GBM, aiming to correlate these profiles with GBM patients’ survival and prognosis. To name but a few, Yuan et al. suggested a 3-miRNA signature (hsa-miR-222, hsa-miR-302, hsa-miR-646) as a predictor of overall survival (OS), utilizing miRNA expression data for GBM patients from the Cancer Genome Atlas (TCGA) dataset [8]. A recently published 4-miRNA signature showed prognostic value, taking into account *MGMT* promoter methylation and age as cofactors, in *IDH1/2* wild type GBM patients [9].

Herein, we report a 3-miRNA signature (hsa-miR-20a, hsa-miR-21, hsa-miR-10a) to map the inter-individual variability of glioblastoma multiforme patients, accounting for confounding factors and selection bias, toward optimum patient stratification. Molecular-clinical correlations were drawn to classify high- (OS < 12 months) vs. low-risk (OS > 12 months) patients, also shedding light on the molecular mechanisms involved in disease progression. We have validated our 3-miRNA signature by quantitative real-time PCR (qRT-PCR) coupled to discriminant analysis. Furthermore, we performed in-silico validation, based on immunohistochemistry (IHC) images of GBM biopsies, employing a hybrid deep learning system [10].

## 2. Materials and Methods

### 2.1. Mixed-Methods Content Analysis

A mixed-methods content analysis was conducted as it is considered the gold standard approach for a content analysis, after the synergy of inductive (qualitative) and deductive (quantitative) phases, especially when it comes to contemporary definitions. Our mixed methods content analysis consisted of mining (data and text) and data analysis. We mined omics datasets, peer-reviewed literature and clinical trial outcomes databases (as of 2021) to explore inter-individual variability in GBM and overall survival datasets. Furthermore, a novel frame-work was developed to meet the aims of our analysis, interrogating data in terms of content and context. We relied on literature data from PubMed/MEDLINE and Scopus, as they are considered the largest abstract and citation databases of peer-reviewed literature. To avoid selection biases, both publicly available and private texts have been assessed (according to our inclusion/exclusion criteria). A series of MeSH terms (www.nlm.nih.gov/mesh, accessed on 23 January 2020) and keywords were used, namely «*GBM OR glioblastoma AND hsa-miR-20a AND biomarker*», «*GBM OR glioblastoma AND hsa-miR-21»*, *«GBM OR glioblastoma AND hsa-miR-10a*». The interim output was questioned further for sample size (validated by a power analysis), open data (yes/no) and research strategy, as well as the impact/metrics of the publication in question (Q1 or Q2: citation index/scientific journal rankings). Studies on non-human samples or those that failed to meet the aforementioned criteria were excluded. The interim and final outputs (*n* = 13) were co-analyzed by two co-authors (V.B. and T.K.), with the percentage of inter-rater agreement calculated. Both percentage agreement and Cohen’s kappa statistic have been calculated to avoid biases (in particular, the possibility that raters guessed on scores) by SAS^®^ macro MAGREE with multicategorical ratings.

### 2.2. Glioblastoma Multiforme Patient Cohort and Clinical Samples

The study protocol is in accordance with the Declaration of Helsinki and has been approved by the ethics review board of the General University Hospital of Patras, Greece. IRB protocol number: 8735/142. Written informed consent was obtained from each individual participating in the study. All patients (*n* = 37) were diagnosed with histologically confirmed GBM, according to the World Health Organization classification of tumors of the central nervous system and were treated by the standard-of-care treatment protocol [11,12]. The demographic and clinical characteristics of our patient cohort are summarized in Supplementary Table S1. OS was calculated from the time of diagnosis until death or last follow-up (12 months).

### 2.3. Immunohistochemical Analysis

Immunohistochemistry for DNA mismatch repair protein MSH2 (MSH2), a validated target for hsa-miR-21, was performed on formalin-fixed, paraffin-embedded (FFPE) GBM samples. anti-MSH2 (1:8000 dilution, ab227941, Abcam plc) was used as the primary antibody. IHC staining was performed according to the manufacturer’s instructions. In brief, sections were deparaffinized in xylene and rehydrated in a graded ethanol series. For heat-induced antigen retrieval, samples were immersed in ethylenediamine tetraacetic acid (EDTA) solution and were then heated in a microwave for 3–5 min and incubated for 40 min at room temperature. Following this, sections were treated with H_2_O_2_ 3% *v*/*v* and washed with Tris-buffered saline (TBS) solution. Sections were then incubated with 3% *w*/*v* blocking solution, bovine serum albumin (BSA) for 15 min, followed by primary antibody reaction. Subsequently, sections were washed five times with TBS and incubated with secondary antibody, using HRP-labelled polymer DAKO EnVision^TM^ (K5007, Dako Glostrup, Denmark), followed by washes with TBS. Staining was visualized using diaminobenzidine, DAB (Dako Company Glostrup, Glostrup, Denmark) and was counterstained with haematoxylin (Haematoxylin Harris Acidified, Atom Scientific, Manchester, UK), dehydrated in ethanol, and cleared in xylene. The slides were cover-slipped using xylene diluted agent (DPX Mountant Low Viscosity, Atom Scientific, Manchester, UK).

### 2.4. MiRNA Extraction

Total RNA was extracted from FFPE tissue using the miRNeasy FFPE Kit (cat. No. 217504, Qiagen, Germantown, MD, USA). Briefly, excess paraffin was trimmed off the sample block and 320 μL Deparaffinization Solution (cat. No. 19093) was added to the sections. Following this, vortexing and brief centrifugation were applied. After incubation at 56 °C for 3 min, 240 μL Buffer PKD and 10μL proteinase K were added to the lower clear phase, followed by incubation at 56 °C for 15 min and then at 80 °C for 15 min to release RNA from the sections studied. The lower, clear phase was incubated on ice for 3 min and centrifuged for 15 min at 13,500 rpm. The DNase digestion step was performed by adding the DNase Booster Buffer in a ratio of 1:10 of the total sample volume to the supernatant and 10μL DNase I stock solution against DNA contamination, including highly fragmented molecules. Following this, 500 μL Buffer RBC was added to the supernatant and the lysate was mixed thoroughly. Absolute ethanol was added to provide the appropriate binding conditions for RNA and 700 μL of each sample were then applied to a RNeasy MinElute spin column, where total RNA, including miRNA, binds to the membrane. Contaminants were efficiently washed away by adding Buffer RPE, following a centrifugation step. The flow-through was discarded. The RPE step was repeated. The RNeasy MinElute spin column was placed in a new 2 mL collection tube and centrifuged at full speed for 5 min. For RNA elution, the RNeasy MinElute spin column was placed in a new 1.5 mL collection tube,14 μL RNase-free water was added and centrifugation was performed for 1 min at full speed.

### 2.5. cDNA Synthesis

miScript II RT Kit (cat. No. 218161, Qiagen, Germantown, Maryland, USA) was used and miScriptHiSpec Buffer was selected to prepare cDNA for the subsequent quantification of mature miRNA. The reverse transcription reaction components in a total volume of 20 μL are listed as followed: 4 μL 5× miScriptHiSpec Buffer, 2 μL 10× miScriptNucleics Mix, 2 μL miScript Reverse Transcriptase Mix plus 7μL RNase-free water 5μL template RNA with concentration 10 ng–2 μg. Following this, an incubation step at 57 °C for 60 min took place, followed by an extra incubation step at 95 °C for 5 min for enzyme inactivation.

### 2.6. Quantitative Real-Time PCR (qRT-PCR)

The quantitative real-time PCR reaction was carried out using miScript SYBR Green PCR Kit (cat. No. 218073, Qiagen, Germantown, Maryland, USA) for the quantification of hsa-miR-20a, hsa-miR-21 and hsa-miR-10a. To normalize the amount of target miRNA, SNORD96A served as the endogenous reference RNA, as it has been verified to have relatively stable expression levels in brain tissue. Each sample was diluted to 200 μL RNAase-free water. The total reaction volume was 25 μL and the reaction setup was as detailed below: 12.5 μL 2× QuantiTect SYBR Green PCR Master Mix, 2.5 μL 10× miScript Universal Primer, 10× miScript Primer Assay (Hs_miR_20a_2 cat. No. MS00003199, Hs_miR_21_2 cat. No. MS00009079, Hs_miR_210a_2 cat. No. MS00031262, Hs_SNORD96A_11 cat. No. MS00033733), 5 μL RNase-free water and 2μL template cDNA. Cycling conditions for qRT-PCR were, as follows: 95 °C for 15 min; 45 cycles of 94 °C for 15 s, 55 °C for 30 s and 70 °C for 30 s. qRT-PCR was performed in the thermal cycler CFX96 TouchTM Real-Time PCR Detection System (BIO-RAD). The relative expression of each miRNA was normalized to the control SNORD96A and ΔCq was calculated by subtracting the Cq of the investigated miRNA from the Cq of the endogenous control.

### 2.7. Statistical Analysis

#### 2.7.1. MiRNA Signature Analysis

Data analysis was performed to reveal any hidden patterns in our GBM cohort. The Shapiro–Wilk test was applied to all the variables of our data (age and miRNA expression) per group to test whether they follow a normal distribution. Subsequently, since we have a small sample size, we performed a Wilcoxon signed-rank test to assess whether the age differs between the low- and high-risk groups. Following this, a chi-square test was performed to assess for sex-dependent differences in our study groups. The expression values from our 3-miRNA signature were also interrogated by a two-way ANOVA to test if there was similar variance between our two groups. Furthermore, the ANOVA was followed by a Wilcoxon signed-rank test for each one of the three miRNAs to determine which miRNAs differentiate between the low- and high-risk group of patients. For the aforementioned analysis we employed the R-language (version 4.1.0).

#### 2.7.2. Survival Analysis

We carried out Kaplan–Meier analyses to calculate the survival probability and visualize the survival curves for the low- and high-risk groups. A log-rank test was implemented to examine if the survival probabilities of the two groups differ significantly. For survival analysis, we employed the Survival [13,14] and the Survminer [15] R packages.

### 2.8. Discriminant Analysis

#### 2.8.1. MiRNA Raw Data

To test in silico whether our 3-miRNA signature can discriminate the low- and high-risk group of patients, a machine learning approach was implemented. Before fitting the miRNA expression values to the models, variable correlation was checked with a Pearson’s correlation coefficient test to assure that the models’ performance will not be affected by the so-called multicollinearity problem [16]. In the discriminant analysis the data were split into low- and high-risk groups and then were used to train five machine learning algorithms. Since our sample size was rather limited, we opted for the bootstrap method [17] for the evaluation of the trained models. To evaluate each predictor in our models, we used the varImp function. The training and validation processes were repeated 10 times. The selected machine learning models employed were: linear discriminant analysis, naïve Bayes, k-nearest neighbors, support vector machines and random forest. Herein, we consider as sensitivity the ability of the models to identify high-risk patients correctly as high-risk. Similarly, specificity is defined as the ability of the model to predict correctly the low-risk patients. Our discriminant analysis was implemented by the caret R-package [18].

#### 2.8.2. IHC Images

A set of n = 45 IHC images were analyzed to cross-validate that low- and high-risk groups can be discriminated based on antibody detection. Hence, we opted for an image analysis method, which would extract representative features from the entire image and not just textural or structural features from a specific area of the image in question. For this, we employed VGG16, a convolutional neural network (CNN) for feature extraction, as proposed in the studies of Xu et al. and Yonekura et al. [19,20]. More specifically, VGG16 is a pretrained CNN model with 16 layers proposed by the Visual Geometric Group [21]. It was trained with ImageNet, which is a dataset of approximately 1.2 million non-medical images, belonging to 1000 classes. Thus, this model has the ability to extract features from a wide range of images and then classify them. To extract features from our IHC images, we resized them to 224 × 224 × 3, which is the VGG16 input requirement. Subsequently, images advanced on the convolutional layers and features were extracted from the 4th pooling layer of the CNN, creating a 100,352-dimensional feature vector for each image. The whole process was performed in R language using Keras [22] and Tensoflow [23] packages.

Following this, we constructed a matrix by binding the feature vectors derived from the images studied. Further processing was required to use those data for discriminant analysis. At first, we excluded the features that had zero values either for all the samples or for the majority of samples (>50%), since they would operate as noise in the meaningful data. Thus, the dimensions of each image vector dropped down to 9532 features. Subsequently, due to the high dimensionality of our matrix, we used principal component analysis (PCA) to reduce the features to an orthogonal matrix. Before the application of the PCA transformation, data were scaled and a set of n = 45 principal components (PCs) was collected. Following this, we employed the recursive feature elimination (RFE) algorithm [24], which is a feature selection technique that would unveil the PCs that discriminate our two groups better. For the implementation of the RFE, we set a threshold to select up to seven features. This threshold was calculated by dividing by three the number of samples that belong to the group with the fewer patients in question to avoid the overfitting of the machine learning models employed. A similar methodology has been also successfully applied by Theodosi et al. [10]. Data classification was carried out first with unsupervised algorithms to see if data can be discriminated into clusters without the model knowing the group they belong to. All *n* = 7 features combinations were tested to find the one that performs best. The unsupervised algorithms that were used in our analysis were the following: k-means, agglomerative clustering, Gaussian mixture and mini batch k-means. We also applied supervised techniques by employing *n* = 10 different classifiers. In the supervised models, we used the features that yield the best performance in the unsupervised learning. The classifiers used were the following: nearest centroid, k-nearest neighbors (k-nn), Gaussian naive Bayes (gaussiannb), linear discriminant analysis (lda), logistic regression, perceptron, multi-layer perceptron, nu-support vector (nusvc), random forest and decision tree. To evaluate the performance of each classifier, the bootstrap evaluation method was repeated 10 times. The same assumptions for specificity and sensitivity as in the discriminant analysis of the expression data were also made for these models. The aforementioned process was implemented in Python3 (version 3.6), Sklearn package [25].

## 3. Results

### 3.1. A Mechanistic View of Our 3-miRNA Signature in GBM

**Figure 1:**
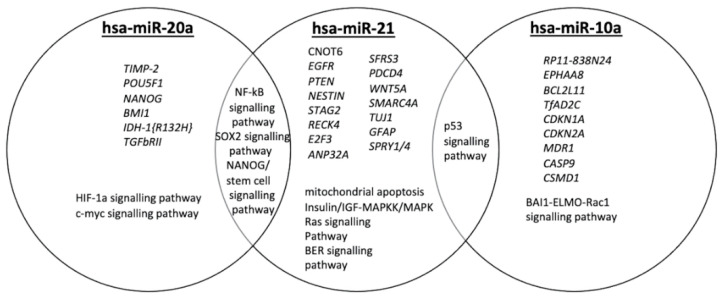
Qualitative Venn diagram of data and text mining findings. Venn diagram representing genes and GBM-related pathways targeted by hsa-miR-20a, hsa-miR-21 and hsa-miR10a.

We identified a subset of validated target genes and cancer-related pathways for each miRNA studied (Figure 1), indicating the biological impact of hsa-miR-20a, hsa-miR-21 and hsa-miR-10a in GBM. We found that these three miRNAs have been associated with key molecular processes in GBM, inter alia, angiogenesis, radiosensitivity, cell migration and invasion [7,26,27]. As depicted in Figure 1, hsa-miR-20a, when overexpressed, promotes angiogenesis, cell invasion and cell growth, while hsa-miR-21 plays a key role in invasiveness, radiosensitivity and/or chemosensitivity. hsa-miR-10a overexpression has been observed in epithelial-to-mesenchymal transformation (EMT). Key molecular pathways may be shared among hsa-miR-20a and hsa-miR-21 or hsa-miR-21 and hsa-miR-10a.

### 3.2. Deciphering Age and Sex Dependence in Age- and Sex-Matched Standard-of-Care Treated Patient Cohort

**Figure 2:**
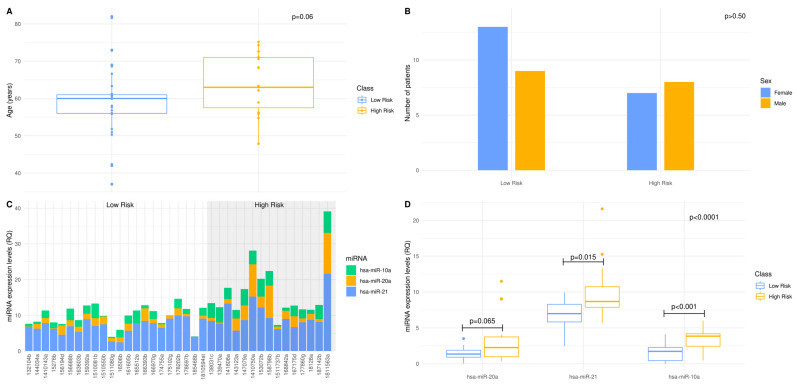
A 3-miRNA signature enables standard-of-care treated patients’ stratification to lower (OS > 12 months) vs. higher (OS < 12 months) risk groups: (**A**,**B**) Distribution of age and sex in low- and high-risk patients of the age- and sex-matched GBM cohort. The 12 months overall survival was used as threshold, to classify the GBM patients into low- and high-risk groups. Statistical analysis was performed by Wilcoxon signed-rank test. There was no statistical significance between low- and high-risk groups regarding age and sex as indicated by *p*-value = 0.06 and *p*-value > 0.50, respectively; (**C**) MiRNAs, hsa-miR-21, hsa-miR-20a and hsa-miR-10a, expression levels in each individual of the standard-of-care treated GBM cohort, as measured by qRT-PCR. The expression of each miRNA was normalized to the endogenous control, SNORD96A. Data are expressed as ΔCq values. The shaded area includes the high-risk group; (**D**) Box and whisker plot for the expression of hsa-miR-21, hsa-miR-20a and hsa-miR-10a in the low- and high-risk group of the standard-of-care treated GBM cohort. The dependence of each miRNA when the patients of the low-risk group are compared to those of the high-risk group, was examined by Wilcoxon signed-rank test. The 3-miRNA signature dependence, when the patients of the low-risk group are compared to those of the high-risk group, was examined by two-way factorial ANOVA for independent samples.

Our findings suggest that 58% of patients are assigned to the low-risk group (OS >12 months) and 42% of the patients to the high-risk group (OS < 12 months), respectively (Supplementary Table S1). Determining the relations among different variables in our GBM cohort, the age values seem to follow a non-normal distribution in the low-risk group of patients, whereas in the high-risk group, they present a normal distribution. As shown in Figure 2A, there was no significant difference between the mean age of the patients with OS > 12 months or the patients with OS < 12 months. When we investigated sex dependence in patients’ stratification into low- and high-risk groups, the proportion of either males or females did not significantly differ between risk groups (Figure 2B). Collectively, our findings indicate that there is no age or sex dependence in our age- and sex-matched standard-of-care treated patient cohort.

### 3.3. The 3-miRNA Signature Expression May Stratify the Standard-of-Care Treated Patients to Lower (OS > 12 Months) vs. Higher (OS < 12 Months) Risk Groups

To assess the prognostic efficacy of our 3-miRNA signature, we determined the expression levels of hsa-miR-20a, hsa-miR-21 and hsa-miR-10a in *n* = 37 FFPE samples of our GBM patient cohort. The differential expression of the 3-miRNAs between GBM samples suggests that we can map and shed light to the inter-individual variability and thus, elucidate individual molecular profiles. It was noted that hsa-miR-21 expression levels were elevated compared with those of hsa-miR-20a and hsa-miR-10a (Figure 2C). Regarding the distribution of each miRNA per risk group, the 3-miRNA expression values in the low-risk group of patients follow a normal distribution, whereas in the high-risk group, only hsa-miR-10a expression values follow normal distribution. The implementation of a two-way ANOVA among the expression values of the 3-miRNAs between the low- and high-risk group yielded a *p*-value of <0.0001. Mean expression between the two risk groups was tested for each miRNA of the 3-miRNA signature, independently. hsa-miR-21 and hsa-miR-10a expression levels were significantly higher in the high-risk group of patients. Meanwhile, no significant difference in the hsa-miR-20a expression levels between risk groups was observed (Figure 2D). Our findings suggest that our 3miRNAs can serve as a patient stratification signature in GBM, as patients can be classified in subgroups of low- (OS > 12months) or high-risk (OS < 12 months).

### 3.4. In Silico Validation of the 3-miRNA Signature

**Figure 3:**
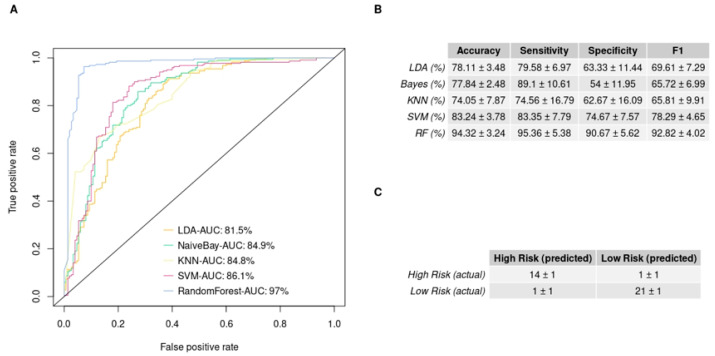
Supervised machine learning algorithms’ performance in discriminating the low- and high-risk group employing miRNA expression values, measured by qRT-PCR: (**A**) The ROC curves of each model predicting the risk group of the validation set. It illustrates the predictive ability that each model has over true positives and false positives. The area under curve (AUC) is also reported; (**B**) The table provides the models and the metrics used for their evaluation (sensitivity is the ability of the models to predict the high risk and specificity the ability to predict the low risk). The table summarizes the mean value and the standard deviation of each mode performance over 10 repetitions of the bootstrap method; (**C**) The mean confusion matrix of the model that scores the best performance on the validation set after 10 repetitions. In the upper left corner of the table, we can see the true positive predictions, and in the lower right, the true negative predictions are depicted.

**Figure 4:**
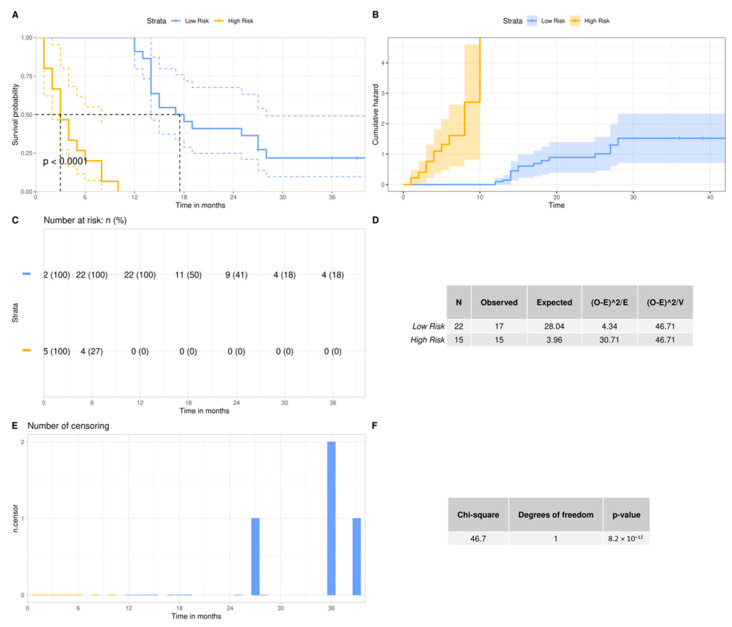
Overall survival probability of the GBM patients according to their risk group**:** (**A**,**C**,**E**) Kaplan–Meier analyses between high- and low-risk group. The patients were stratified to the aforementioned groups, according to their overall survival. In the *y*-axis we can see the proportion of patients surviving and the vertical drops in the curve indicate the events. The number of patients at risk and the number of censorings are supplementary to the survival curve; (**B**) The cumulative events are depicted and indicate the cumulative hazard probability. This is comparable to the integration of the hazard rate of each patient over months; (**D**,**F**) Log Rank test for the comparison of the survival curves of the two risk groups. The null hypothesis in this test is that the two curves do not differ. The fact that our *p* value is below 0.05 indicates that the survival curve of the low-risk group differs significantly from the survival curve of the high-risk group.

To further evaluate the ability of our 3-miRNA signature to stratify our patient cohort, we performed an in-silico validation. We trained five machine learning algorithms with the 3-miRNA expression values. Subsequently, we evaluated their efficiency in discriminating GBM patients into the low- and high-risk groups studied. Figure 3 illustrates the performance of the five algorithms in the validation data. The random forest algorithm yielded the highest performance after 10 repetitions of the training and evaluation process. The random forest algorithm resulted in a mean accuracy of 94.32% with a standard deviation of 3.24%, a mean F1 score of 92.82% with a standard deviation of 4.02% and an AUC of 97% in the validation set. The mean importance of each variable contribution to model predictions was assessed as following: hsa-miR-20a = 9.41, hsa-miR-21 = 7.02, hsa-miR-10a = 13.02. All predictions made on the training data after 10 repetitions of training and evaluation with the bootstrapping method are depicted in Supplementary Figure S1, according to which random forest was the algorithm that outperformed all (100% accuracy, sensitivity, specificity, F1 score and AUC in the training data). This is due to the medium rate of multicollinearity among the three miRNAs, since the Pearson correlation showed that the hsa-miR-20a and hsa-miR-21 were 67% correlated, hsa-miR-20a and hsa-miR-10a were 51% correlated and hsa-miR-10a and hsa-miR-21 were 58% correlated. As random forest was not affected by multicollinearity, it had the best performance, and was also devoid of overfitting. Taking into consideration the significant difference of the survival probabilities of the two groups, as the results of the Kaplan–Meier analysis (*p*-value < 0.001) and the log rank test (*p*-value < 0.0001) indicate in Figure 4, we could infer that the reverse is also feasible. The model is able to efficiently classify patients in the two risk groups by learning their 3-miRNA expression values.

### 3.5. GBM IHC Images Can Be Discriminated into Two Groups, Depending on Overall Survival

**Figure 5:**
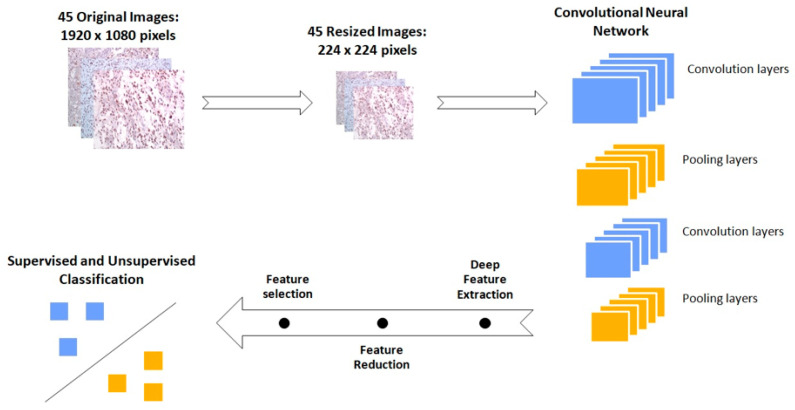
Schematic representation of the workflow followed for the discrimination of the immunohistochemical (IHC) images between low- and high-risk biopsies. The initial images are resized in order to fit in the Vgg16 Convolutional Neural Network. This pretrained network performs the feature extraction. Subsequently the image features are processed in order to be classified from both supervised and unsupervised algorithms.

Feature extraction applied to the IHC images through the CNN produced thousands of features which were transformed to *n* = 45 PCs after PCA analysis. Out of the *n* = 45 PCs, the RFE algorithm showed that those that can better discriminate our two groups, were the 2nd, 3rd, 4th, 5th, 6th, 8th and 38th. Hence, we used only those PCs to train both unsupervised and supervised models (Figure 5).

**Figure 6:**
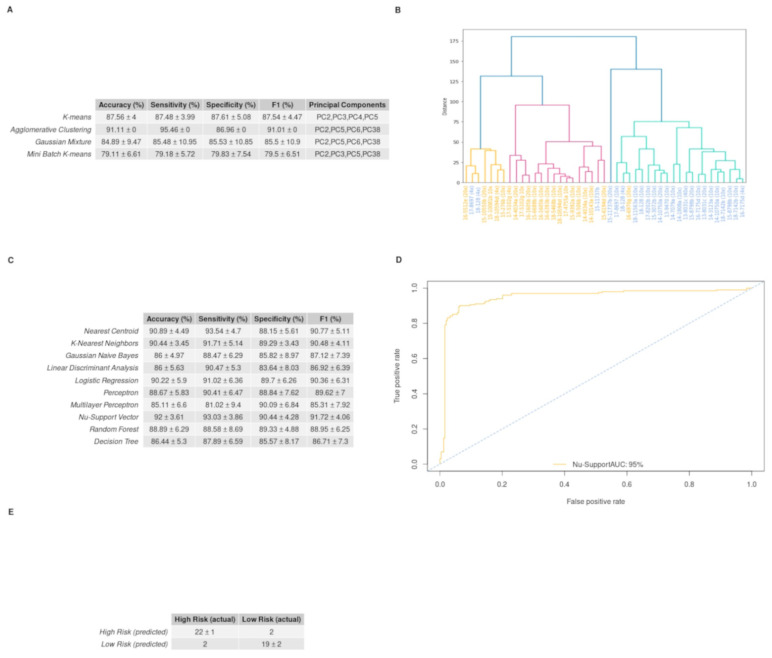
Unsupervised and supervised machine learning algorithms’ performance on discriminating the low- and high-risk groups employing the immunohistochemical (IHC) stained images from GBM biopsy material: (**A**) Dendrogram produced from hierarchical clustering employing Euclidian distance and Ward’s linkage for the selected principal components. On the *x*-axis, the samples of the high-risk images are colored blue, while the samples of the low-risk images are colored orange. The 1st cluster, which consists of the orange and the red groups, represents the low-risk cluster, while the turquoise group along with the 22nd (15-11737b (20×)) sample constitutes the high-risk cluster (sensitivity is the ability of the models to predict high risk and specificity the ability to predict low risk); (**B**) The ROC curve of the supervised model, with the best performance in discriminating the two groups, after 10 repetitions of the bootstrap method. The area under the curve is equal to 0.96; (**C**) The table presents the unsupervised models and the measurements used for their assessment. It contains the mean value, the standard deviation and the feature combination of each model that yield the best performance over 10 repetitions of the bootstrap method (as sensitivity is the ability of the models to predict the high risk and as specificity the ability to predict the low risk); (**D**) The table shows the performance of the 10 supervised algorithms, in the validation set, after 10 repetitions of the evaluation method. The features used for training of the algorithms were PC2, PC5, PC6 and PC38; (**E**) The mean confusion matrix of the Nu-Support Vector model that yields the highest accuracy on the validation set after 10 repetitions.

Optimum performance was achieved by the agglomerative clustering algorithm, which reached an accuracy score of 91.11%. Euclidean distance was set as a measure of distance and Ward’s method as the linkage criteria to specify the dissimilarity between clusters. Standard Deviation (STD) values for accuracy, sensitivity and specificity were equal to zero, as it is not a stochastic algorithm. The PCs that led to this result were the 2nd, 5th, 6th, and 38th. Figure 6A depicts how each patient was clustered in a dendrogram plot. Agglomerative clustering, like all the hierarchical clustering methods, works from bottom up by merging the nearest clusters at each step, until there is only one cluster containing all the samples of the dataset [28]. The results of our analyses were initially organized in four clusters: the turquoise, the red, the orange and the cluster containing only the 22nd sample. Following this, two clusters were obtained: one for the orange and the red sub-clusters and another one for the two remaining clusters. In the dendrogram it is apparent that the first cluster represents the low-risk group with four misclassified samples, while the second one represents the high-risk group with only one misclassified sample. Thus, sensitivity rises to 95.46%, specificity to 86.96% and F1 score to 91.01%. The performance of all the clustering algorithms is depicted in Figure 6C. The distribution of the four PCs that yielded the highest accuracy is depicted with box plots in Supplementary Figure S2. Those PCs were also used to train *n* = 10 supervised algorithms. The performance of each model on the validation set is provided in Figure 6D. The highest mean accuracy score of 92.00% with 3.61% standard deviation was achieved by the Nu-Support Vector classifier and a mean F1 score of 91.72%, with 4.06% standard deviation obtained in the validation set after 10 repetitions. This model’s performance is also illustrated with its ROC curve in Figure 6B, with an AUC = 95%. The mean confusion matrix for this model is given in Figure 6D. The model classifies patients from both groups.

## 4. Discussion

GBM patients are a remarkably heterogenous group, presenting different prognoses for OS. Such intra-individual heterogeneity of GBM requires more effective biomarkers from the perspective of clinical implementation, which will be accomplished by the integration of individual molecular profiling together with a better understanding of the disease phenotype. Herein, we assess the prognostic value of a 3-miRNA signature that allows GBM patient stratification into low- and high-risk groups.

Following our mixed-methods content analysis, the key role for hsa-miR-20a, hsa-miR-21 and hsa-miR-10a was revealed in the regulation of gene expression in GBM pathophysiology. The functional role of hsa-miR-20a, which is found overexpressed in GBM, based on its validated targets TIMP-2, TGFb-RII and CTGF, is associated with increased cell invasion, angiogenesis and cell growth [26,29,30]. The upregulation of hsa-miR-21 has been correlated with reduced cellular radiosensitivity and chemosensitivity, through its implication in DNA-repair mechanisms and cell-cycle linked pathways [7]. hsa-miR-10a overexpression has been linked to increased cell invasion and migration, as it regulates epithelial-to-mesenchymal transformation (EMT) [27]. Our findings reveal the oncogenic function of the abovementioned miRNAs, as patients with unfavorable prognoses have been associated with higher miRNA expression levels, compared with those with better prognoses. It is of note that slightly lower expression values of hsa-miR-10a may be attributed to upstream negative regulators, such as the long non-coding RNA TUSC7 [31].

Several studies have shown that distinct miRNA expression patterns may serve as diagnostic, prognostic or predictive candidate biomarkers. To name but a few, a GBM miRNA profile, including nine differentially expressed miRNAs in FFPE samples, could discriminate GBM from gliomas of grades I-III [32]. Moreover a 4-miRNA signature has been proposed that can determine short- and long-term survival in GBM patients [33]. Additionally, the prognostic value of miRNA signatures and their OS prediction ability have been reported in various studies [34,35,36,37,38]. Weighing up the aforementioned studies, our 3-miRNA signature consists of a unique, low complexity combination of miRNAs and may be applied regardless of confounding variables, such as MGMT status or GBM molecular subtype.

In the present study, miRNA expression analysis was performed on a balanced GBM cohort. The FFPE material used was that of choice as it is the most reliable source for miRNA isolation and the most standardized way to process tissue in a clinical routine [32]. For miRNA quantification, we used qRT-PCR, as the gold-standard methodology, characterized by high sensitivity and specificity [39]. Importantly, our findings show that we can extract a distinct miRNA expression profile for each individual, which reflects disease severity. To avoid selection biases, considering the sample size of this study, instead of a population-based estimate, 12 months was the cut-off value set for the low- and high-risk groups, as reported by pivotal studies (RT+TMZ) in GBM and/or clinical trials for newly diagnosed patients and the age-groups included herein [40,41,42]. Our analysis revealed a clear tendency of higher expression levels for our 3-miRNA signature in the high-risk group.

Our 3-miRNA signature expression profile discriminates the two prognostic groups. Among the three miRNAs, although differences do exist, hsa-miR-20a expression has no significant difference between the risk groups. This result may be attributed to the specific sample space, upon consideration of the established oncogenic function of hsa-miR-20a, its implication in gliomagenesis and the meta-analysis findings, which have shown its association with an increased hazard of death [43]. To our knowledge, only two studies have reported on hsa-miR-20a as a protective miRNA in GBM [29,44].

Our in-silico validation by coupling machine learning to a pathomics approach empowers our 3-miRNA signature value as a useful tool for risk stratification in GBM. Machine learning algorithms, using the 3-miRNA expression values, show a very good predictive ability for high-risk (sensitivity = 95.36%) and low-risk (specificity = 90.67%) patients. We note that despite hsa-miR-20a expression levels not being significantly different between the two groups, it is effectively used as a predictor in our models (mean importance of hsa-miR-20a = 9.41), indicating that the combination of those miRNAs as a signature can discriminate patients into high- and low-risk groups, rather than each miRNA alone. The fact that the algorithm seems to be in favor of the high-risk group according to our view rises from the low variability that our 3-miRNA signature has in the high-risk group. Despite there was no external test set available for further evaluation of the models’ generalization, the random forest classifier produced robust predictions. This was reflected in the low standard deviation in its accuracy among the 10 iterations of the models’ design and evaluation. As our model does not overfit, we conclude that the prediction of patients’ risk groups from an independent GBM cohort, providing the 3-miRNA expression profile, is feasible. In this context, we used the mean predictions made by the random forest to perform the Kaplan–Meier analysis (graphs not shown). The survival probabilities produced the same results as the Kaplan–Meier analysis which was done based on the OS and thus these predictions could be eventually used to get the survival probabilities of an independent cohort. Our hybrid deep learning model, based on the GBM IHC images, classifies the patients from both groups quite well. There are several studies performing pattern recognition in different modalities of GBM images [45,46,47]. In most of the cases, images were used to discriminate GBM molecular features or subtypes, whereas we aim to stratify patients into low- and high- risk groups. The PCs used in the machine learning process have a rather low variance, which is not considered optimal; however, as Jolliffe [48] suggests, PCs with a low variance can also be used as predictors. Indeed, in our case PCs were able to discriminate the two groups, which verifies the significance of our 3-miRNA signature toward patient stratification.

GBM constitutes a major challenge in selecting the most effective therapeutic approach due to tumor heterogeneity, inter-individual variability, late diagnosis and poor prognosis, as well as limited therapeutic options. Since miRNAs exhibit a pivotal role in glioma pathophysiology, miRNA profiles present information-rich signatures and may serve as a toolbox towards optimum disease management. We and others working on better-informed decisions and translational biomarkers call for a non-stop critical appraisal of study design and data reliability. There is no “one-size-fits-all.” Multivariate analyses that also include the *IDH* mutation status, *MGMT* promoter methylation status and Verhaak GBM subtypes may corroborate independent prognostic predictors rather than being an epiphenomenon of established prognostic markers. Herein, a prospective study was designed with extra care for the patient cohort to be as fully characterized as possible—not accidentally homogenous, in an attempt to account for confounding factors and overcome selection bias. First, the choice of a single clinical site ensures the data quality of, for instance, TMZ cycles, in particular, are difficult to ascertain from data files or retrospective studies. Following this, we chose not to use multivariate regression methods as the means against selection bias, but our thorough hybrid deep learning approach, instead. For sure, there are several prognostic factors with regard to survival that are not registered, i.e., unmeasured confounders [49], that play a crucial role in predicting OS in addition to those mentioned above: performance status, extent of resection, and Mini-Mental State Examination [50]. So far, none of these factors had a hazard ratio over 4. In a 5-year follow-up of TMZ data, neither recursive partitioning analysis class nor *MGMT* promoter methylation have hazard ratios of such magnitude [51]. Helseth et al. (*n* = 66) reported a significant association between OS and patients with *MGMT* promoter methylation status and the extent of resection with hazard ratios of 7.9 and 4.5, respectively [52]. We advise that a sensitivity analysis based on the methodology detailed by Rosenbaum is required to demonstrate the impact necessary for any unmeasured confounder to invalidate findings [53]. In our study, any confounder not controlled for would need an odds ratio of at least 4 to invalidate our findings, also validated by a hybrid deep learning approach.

Future perspectives cannot but aim at innovative strategies with potential impacts in clinical practice [54,55].

## 5. Conclusions

GBM is a rather complex disease trait that renders optimum decision-making hard, in particular when the poor survival rate and therapeutic options are considered. To this end, miRNAs serve as a toolbox toward biomarker discovery. We herein suggest the synergy of wet- and dry-lab approaches as a viable solution to the deleterious issue of translational biomarkers, which are most hampered by poor data quality and biases. Our pipeline accounts for confounding factors in both test and validation phases. Our 3-miRNA signature (hsa-miR-20a, hsa-miR-21, hsa-miR-10a) is able to stratify GBM patients and hence may contribute to OS prediction to empower evidence-informed decision-making in clinical practice and enrich drug repurposing pipelines.

## Data Availability

All data generated or analyzed during this study are included in this published article and its supplementary information files.

## Supplementary Materials

The following supporting information can be downloaded at: https://www.mdpi.com/article/10.3390/curroncol29060345/s1, Figure S1: Supervised models performance on the training set for the discriminant analysis of the qRT-PCR data in low- and high-risk group: (A) ROC curves and the corresponding AUC for each model predictions on the data that were used for the training of the classifiers. The Random Forest algorithm results in the highest AUC. (B) The mean value and standard deviation of the metrics used to evaluate the five machine learning algorithms for 10 iterations.; Figure S2: Boxplots of the Principal Components combination that gives the best discrimination between the low- and high-risk group: (A,B,C) The 2nd, the 5th and the 6th Principal Components differ statistically among the 2 groups. (D) The 38th Principal Component does not differ statistically between the two categories.; Table S1: Demographic and clinical characteristics of the standard-of-care treated GBM patients.
