# Supplementary material for: A 3-miRNA Signature Enables Risk Stratification in Glioblastoma Multiforme Patients with Different Clinical Outcomes
Source: Curr Oncol. 2022 Jun 16;29(6):4315–31. doi: 10.3390/curroncol29060345 (PMC9221847; doi:10.3390/curroncol29060345)
Supplement: Supplementary file 1 [file curroncol-29-00345-s001.zip › curroncol-1728315-supplementary.pdf]

Table S1. Demographic and clinical characteristics of the standard-of-care treated GBM patients.

Characteristic	GBM cases (n = 36)
Sex	
Female	19 (53.00%)
Male	17 (47.00%)
Caucasian	36 (100.00%)
Age at surgery/biopsy [years]	
Median	61
Range	37–82
Overal Survival [months]	
Median	13.50
Range	45–1
OS Risk Group	
Low Risk Group [OS>12 months]	21 (58.00%)
High Risk Group [OS<12 months]	15 (42.00%)

A

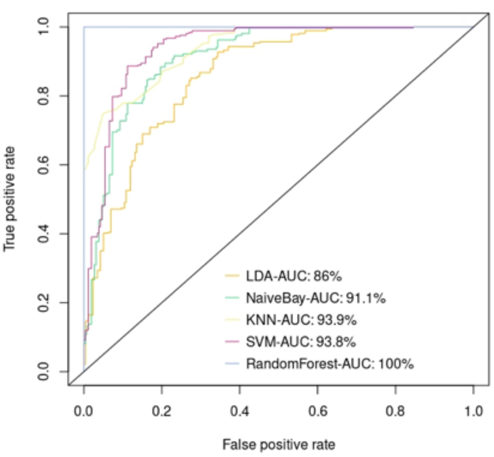

B

	Accuracy	Sensitivity	Specificity	F1
LDA (%)	79.84 ± 6.48	82.86 ± 8.46	65.93 ± 12.85	72.48 ± 8.97
Bayes (%)	82.06 ± 3.75	94.3 ± 7.79	60.72 ± 7.44	73.3 ± 4.76
KNN (%)	83.49 ± 4.25	84.15 ± 9.98	75.22 ± 15.56	77.92 ± 7.56
SVM (%)	89.21 ± 5.06	92.31 ± 6.2	80.74 ± 10.85	85.66 ± 6.22
RF (%)	100 ± 0	100 ± 0	100 ± 0	100 ± 0

Figure S1. Supervised models performance on the training set for the discriminant analysis of the qRT-PCR data in low- and high-risk group: (**A**) ROC curves and the corresponding AUC for each model predictions on the data that were used for the training of the classifiers. The Random Forest algorithm results in the highest AUC. (**B**) The mean value and standard deviation of the metrics used to evaluate the five machine learning algorithms for 10 iterations.

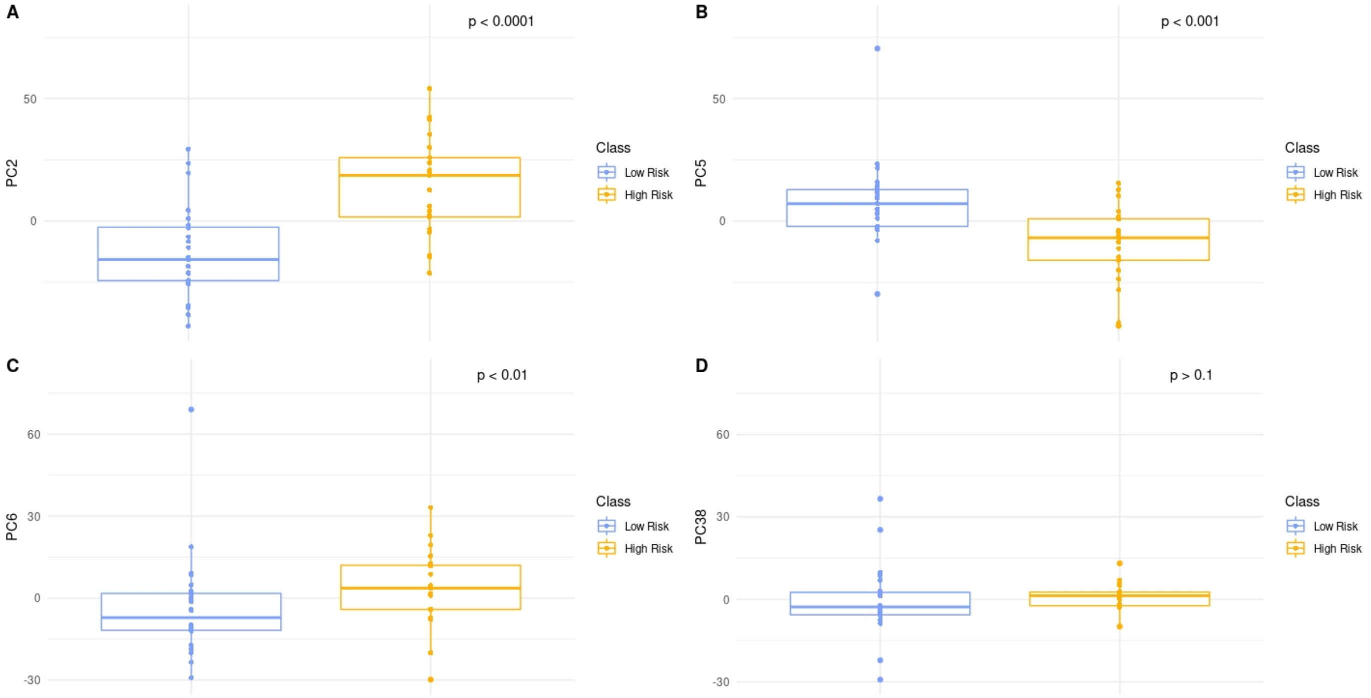

Figure S2. Boxplots of the Principal Components combination that gives the best discrimination between the low- and high-risk group: (**A**,**B**,**C**) The 2nd, the 5th and the 6th Principal Components differ statistically among the 2 groups. (**D**) The 38th Principal Component does not differ statistically between the two categories.
